# Introduction of barley to the Tibetan Plateau: an important step toward Tibetan civilization

**DOI:** 10.1093/nsr/nwz139

**Published:** 2019-09-16

**Authors:** Song Ge

**Affiliations:** State Key Laboratory of Systematic and Evolutionary Botany, Institute of Botany, Chinese Academy of Sciences, China

Domestication is generally defined as a distinctive co-evolutionary, mutualistic relationship between domesticator and domesticate [1] and represents a critical evolutionary transition in human history with profound and lasting global impacts [1,2]. Crop domestication consists of numerous outstanding examples of coevolution between humans and plants because ancient people completed the domestication of all major crops by 4000 years ago [3]. In recent decades, the advent of new archaeological, genetic and genomic techniques has significantly improved our understanding of the origin of domestication and agriculture in general. However, the spatial and temporal patterns of domestication, particularly the interface between humans and plants, remain less investigated. In this issue of *NSR*, based on archaeological and high-resolution genetic data, Li *et al.* [4] present a state-of-the-art investigation on the history of early human settlement on the Tibetan Plateau (TP), providing new insights into the origin and settlement of Tibetans.

Although the TP, also called the Qinghai-Tibet Plateau or Himalayan Plateau, is considered inhospitable to humans, multiple lines of evidence have shown that the TP has been inhabited since the late Upper Pleistocene [5,6] and that permanent human occupation in the plateau began approximately 3600 years ago and was likely facilitated by the introduction of cold-tolerant barley agriculture [5,7]. Although it remains unknown who brought this exotic crop to the TP, two hypotheses are available, i.e. the barley was introduced to the TP either by millet farmers from lower elevations in the northeastern TP or by immigrations from West Asia [7]. By exploring tens of thousands of mitogenomes in Tibetans and surrounding populations, including those who descended from Neolithic millet farmers, in cooperation with radiocarbon dating of cereal remains from archaeological sites on the TP and in northern China, Li *et al*. [4] showed that two mitochondrial haplogroups in current Tibetans could trace their ancestry back to northern China approximately 10 000 years ago and have *in situ* differentiation on the TP 5200–4000 years ago, representing the genetic legacy of Neolithic millet farmers in contemporary Tibetans. The finding of a substantial genetic contribution by the Neolithic millet farmers to the Tibetan gene pool at the time of barley dispersal to the TP provided the first piece of evidence demonstrating that it was Neolithic millet farmers from the lower-altitude northeastern parts of the plateau, rather than the immigrants from West Asia, who brought barley agriculture to the TP approximately 3600 years ago and contributed to permanent human settlement on the plateau.

As nicely shown by Li *et al*. [4], genetic information arising from high-resolution data, in combination with archaeological evidence, is able to unravel scenarios that seem elusive. Despite this progress, as the authors acknowledged [4], investigations based on large-scale and high-resolution Y chromosome data are needed to better understand the migration history of Tibetans. On the other hand, knowledge of plant and animal domestications would help the understanding of the history of agricultural development and human civilizations. In these contexts, it is still premature to draw a full picture of Tibetan origin and settlement before a few questions are convincingly addressed, including the exact interaction between different groups of people on the TP and in the surrounding areas, how the millet farmers adopted barley agriculture after their arrival to the northeastern TP and the process of dispersal and cultivation of barley (and perhaps some other domesticated plants and animals) on the plateau. From the viewpoint of domestication, it would also be interesting to ask how the new settlers of crops and livestock, such as barley and sheep (a cold-tolerant livestock), adapted to the extreme and harsh environment on the TP and responded to varying levels of human manipulation. To solve these questions is directly relevant to efforts to improve existing crops and livestock and introduce new species with potential value for domestication and utilization as genetic resources [1,2].

## References

[1] Zeder MA . Proc Natl Acad Sci USA 2015; 112: 3191–8. 25713127 10.1073/pnas.1501711112 PMC4371924

[2] Larson G , Piperno DR and Allaby RG et al. Proc Natl Acad Sci USA 2014; 111: 6139–46. 24757054 10.1073/pnas.1323964111 PMC4035915

[3] Doebley JF , Gaut BS and Smith BD et al. Cell 2006; 127: 1309–21. 17190597 10.1016/j.cell.2006.12.006

[4] Hein A . Archaeology of Tibetan Plateau. In: Smith C (ed.). Encyclopedia of Global Archaeology. Switzerland AG: Springer Nature, 2018.

[5] Li Y-C , Tian J-Y and Liu F-W et al. Natl Sci Rev 2019; 6: 1005–13. 10.1093/nsr/nwz080 PMC8291429 34691962

[6] Zhang XL , Ha BB and Wang SJ et al. Science 2018; 362: 1049–51. 30498126 10.1126/science.aat8824

[7] Chen FH , Dong GH and Zhang DJ et al. Science 2015; 347: 248–50. 25593179 10.1126/science.1259172

